# Neurobiology Research in Parkinson’s Disease

**DOI:** 10.3390/ijms21030793

**Published:** 2020-01-25

**Authors:** Takao Yasuhara

**Affiliations:** Department of Neurological Surgery, Okayama University Graduate School of Medicine, Dentistry and Pharmaceutical Sciences, 2-5-1, Shikata-cho, Kita-ku, Okayama-shi, Okayama 700-8558, Japan; tyasu37@cc.okayama-u.ac.jp; Tel.: +81-86-235-7336

In recent years, therapeutic strategies for Parkinson’s disease (PD) have been developing in many different ways. In addition to the medication, surgery, rehabilitation, and other established therapies, various options using cell therapy, electrical stimulation, and neurotrophic agents are becoming a reality. The pathological condition of PD, animal model making, and mechanisms to progress disease status and to exert therapeutic effects by each treatment should be synergistically explored in basic researches. In this Special Issue, various topics on neurobiology research in PD are described including the pathological condition, disease progression, and future therapeutic options of PD. Daily administration of pomegranate juice enriched in ellagitannins exerted neuroprotective effects in the rotenone rat model of PD through anti-apoptotic effects related to anti-oxidative potentials [1]. Well-known food or drugs might have some neuroprotective potentials on various pathological conditions in the central nervous system. Gender difference in dopamine deficiency phenotype in the MitoPark mice model of PD was also explored [2]. Dopamine secretion and expression of tyrosine hydroxylase were strongly affected by gender, that is, female MitoPark mice showed higher preservation of dopaminergic potentials than those of male MitoPark mice. The female predominance was cancelled by ovariectomy, highlighting the need to re-consider neuroprotective potentials of estrogen. Alfa-synuclein aggregation is one of the most important factors in pathogenesis of PD. Disaggregation of alfa-synuclein might be an effective therapy for PD. Peucedanocoumarin III administration resulted in reduced dopaminergic neuronal loss in the 6-hydroxydopamine mice model of PD [3]. The demonstrated neuroprotective effects and safety of peucedanocoumarin III showed the possibility of the clinical application. Neuroinflammation is also a critical response in the pathogenesis of PD. Recently, anti-inflammatory effects of (E)-2-methoxy-4-(3-(4-methoxyphenyl) prop-1-en-1-yl) phenol, a STAT3 inhibitor, was demonstrated. Oral intake of (E)-2-methoxy-4-(3-(4-methoxyphenyl) prop-1-en-1-yl) phenol for 1 month showed strong neuroprotective effects in the 1-methyl-4-phenyl-1,2,3,6-tetrahydropyridine mice model of PD [4]. The control of inflammation is a hopeful therapeutic target for PD. The pathogenesis and disease progression of PD is complex and multifactorial. Related to inflammation, the interactions between brain-gut microbiota are recent topics [5]. Dysbiosis in the gut might be strongly related to systemic and brain inflammation with progression of PD. Vagal nerve stimulation and other approaches for peripheral nerve or systemic circulation might affect the disease progression. In addition to inflammation, alfa-synuclein, mitochondria and endo-lysosomal system interact with each other [6]. The synergistic regulation of each system is also a therapeutic potential for PD. L-DOPA administration is an established medication for PD patients. However, the mechanisms of L-DOPA-induced dyskinesia and other side effects are still unknown and controversial. The review on ‘false’ neurotransmitter of L-DOPA illustrates the complexity of its mechanism of action, which involves serotonergic neurons, L-DOPA itself, and perhaps the so-called trace amines. The review expands our understanding of L-DOPA action and therapy [7]. MicroRNAs play an important role in RNA silencing and post-transcriptional regulation of gene expression. In PD, there are several dysregulated microRNAs including miR-30, miR-29, let-7, miR-485 and miR-26 [8]. In the future, drug discovery and early diagnosis might arise increasingly by the development in microRNAs-related technology. In order to explore mechanisms and new therapeutic strategies for PD, animal models are necessary. Recently, genetic models are increasing, but models induced by neurotoxin are dominant [9]. Most importantly, researchers and readers should know the advantages, disadvantages and limitations of each animal model.

The presented studies in this Special Issue are constructive and hopeful to reveal the underlying neurobiological mechanisms in PD, and to develop new therapeutic options for PD. However, at the same time, clinical research to elucidate the pathological conditions, treatment outcomes, and newly developed therapies are critically important, because there is no small difference between basic researches and clinical settings. We, neuroscientists, neurologists, and neurosurgeons should consider the accumulated results of basic researches on clinical settings in PD, and vice versa, clinical results might deepen the scope for basic researches.
